# Foot Muscle Cross-Sectional Area and Recurrent Falls: A Preliminary MRI-Based Evaluation

**DOI:** 10.7759/cureus.91776

**Published:** 2025-09-07

**Authors:** Atta Taseh, Colin O'Neill, Raveena Joshi, Jennifer Skolnik, Ara Nazarian, John Kwon, Zachary E Stewart, Soheil Ashkani-Esfahani, Adam Tenforde

**Affiliations:** 1 Foot and Ankle Research and Innovation Laboratory (FARIL), Department of Orthopedic Surgery, Massachusetts General Hospital, Harvard Medical School, Boston, USA; 2 Musculoskeletal Translational Innovation Initiative, Carl J. Shapiro Department of Orthopedic Surgery, Beth Israel Deaconess Medical Center, Harvard Medical School, Boston, USA; 3 Musculoskeletal Imaging and Intervention, Department of Radiology, Mass General Brigham, Harvard Medical School, Boston, USA; 4 Department of Physical Medicine and Rehabilitation, Spaulding Rehabilitation Hospital, Harvard Medical School, Cambridge, USA

**Keywords:** fall risk, fall screening, falls rehabilitation, recurrent falls, risk factors of falls

## Abstract

Objective: Foot muscle strength reduction is common in older adults experiencing falls, yet the link between foot muscle radiological characteristics and fall recurrence remains unexplored. This study investigated the association between foot muscle cross-sectional area (CSA), as quantified through MRI, and a history of recurrent falls.

Materials and methods: A retrospective case-control study included patients aged ≥60 years with a history of repeated falls (≥2) and age- and sex-matched controls without falls. Foot muscle CSA at the first tarsometatarsal joint was measured on T1-weighted (n=8) or proton density (n=18) MRI images. Measurements were performed by two independent raters, with inter-rater agreement assessed via the intraclass correlation coefficient (ICC). Multiple regression analysis was performed, adjusting for potential confounding variables. Statistical significance was set at P < 0.05.

Results: Thirteen case subjects with recurrent falls and 13 age-sex matched controls were included. The baseline demographic and physical characteristics were comparable between groups except for foot muscle CSA, which was significantly smaller in the case group (P = 0.006). Regression analysis revealed that a decrease in foot muscle CSA significantly increased the likelihood of recurrent falls, independent of other variables (odds ratio =0.99, 95% CI: (0.98, 0.99), P = 0.03). The interrater agreement was calculated to be excellent (ICC = 0.92, P < 0.01)

Conclusion: Reduced foot muscle CSA may be associated with an increased risk of falls in older adults. This feasibility study lays the foundation for future work investigating fall-risk stratification using this relatively simple quantitative imaging technique.

## Introduction

One in four individuals aged 65 and older experiences a fall annually [1]. Among all falls, approximately 40% are recurrent [2]. Falls account for 2.8 million emergency department visits and approximately US$30 to US$50 billion in medical costs annually [3]. Prior work has characterized risk factors for falls [3,4]. Decreased upper and lower extremity muscle strength is one of the important factors that, if identified and addressed properly, can help improve prevention strategies and reduce the broader societal impact of falls [5].

Aging has been associated with a progressive decline in muscle strength, a term referred to as sarcopenia, which is particularly pronounced in older adults who experience falls [5,6]. A systematic review of 13 studies concluded that lower extremity weakness significantly increases the risk of falls in older adults, including a 76% higher likelihood of any fall and a 206% higher likelihood of recurrent falls [7]. Other studies showed altered balance and functional ability and increased likelihood of falls in those individuals with toe deformities [8-10]. Specifically, decreased toe plantarflexion strength, reduced ankle mobility, hallux valgus, and lesser toe deformities have been associated with an increased risk of falls in the elderly [8,10,11]. Several direct and indirect measures of foot muscle strength have been described, although subjectivity in direct measures may limit their precision and accuracy. A growing body of literature has demonstrated a link between quantitative imaging characteristics of foot muscles, including cross-sectional area (CSA), and their function and strength. A prospective study in 2014 including 26 healthy young adults, found a correlation between measured toe strength and CSA of the intrinsic foot musculature as measured by magnetic resonance imaging (MRI) [12]. However, the feasibility of MRI CSA as a quantitative surrogate for strength in older individuals and as an independent predictor of fall risk has yet to be explored.

The purpose of this study was to evaluate the association of retrospective MRI- derived muscle CSA in older adults ≥60 years with the risk of recurrent falls. We hypothesized that older adults with a history of recurrent falls would have a smaller CSA of intrinsic foot musculature relative to older adults without a history of falls.

## Materials and methods

Study design

A retrospective case-control study was conducted using data from a tertiary hospital, spanning the years 2015 to 2024. Ethical committee approval was obtained, allowing a waiver of participant consent.

Study population

Adults aged ≥60 years with a documented history of more than two falls were identified using the International Classification of Diseases, 10th Revision (ICD-10) code for repeated falls (R29.6), through the institution’s clinical data repository. To confirm eligibility, a comprehensive chart review was conducted to verify a consistent history of recurrent falls and to apply exclusion criteria. Individuals were excluded if they had chronic neurological conditions affecting balance and gait (e.g., multiple sclerosis), diabetic neuropathy, altered foot anatomy due to prior trauma, plantar muscle edema, or MRI reports suggestive of a malignant plantar mass. Eligible participants also had to have undergone a foot MRI within one year of the fall incident. Control subjects were identified using the same data source, excluding any individuals with the repeated falls ICD-10 code or documented history of falls. Controls were also screened to ensure the absence of the same exclusionary comorbidities. Cases and controls whose MRI scans lacked coronal views, did not include the first tarsometatarsal joint, or did not include Proton Density or T1-weighted sequence, were also excluded. A 1:1 ratio matching by age and sex was done between the groups to reduce confounding effects associated with these variables.

Data collection

The initial search identified 733 cases and 141 control patient records. Each was screened by two orthopedic researchers. MRI scans were reviewed by a senior orthopedic researcher, and eligible images were de-identified and retrieved in standard Digital Imaging and Communications in Medicine (DICOM) format from electronic health records for CSA assessments. Demographic and clinical data were extracted from electronic health records for all eligible patients. Demographic variables included age, sex, body mass index (BMI), and race. Clinical variables potentially influencing muscle quality included polypharmacy (defined as the use of ≥5 medications), use of walking aids (canes, walkers, walking frames, or rollators), history of lower extremity vascular disease, and lower extremity pain three months prior to the MRI date [13-16].

MRI measurements**

Foot muscle CSA measurements were performed separately by two orthopaedic researchers, who were not blinded to the diagnoses, using OsiriX Lite Version 14.1.1 (Pixmeo SARL, Bernex, Switzerland). The included images were acquired using a heterogeneous mix of MRI scanners. Most of these were performed on 3T (n=8 per group) and 1.5T (n=4 per group) scanners, though two were performed on low-field 1.16T open scanners (n=1 per group). Slice thickness was 2.5 mm (n=7) or 3 mm (n=17) for all included cases, except for two that were performed at 4 mm. Most of the cases were performed with a field-of-view between 120 and 140 mm, except for two (one at 150 mm and one at 165 mm).

**Figure 1:**
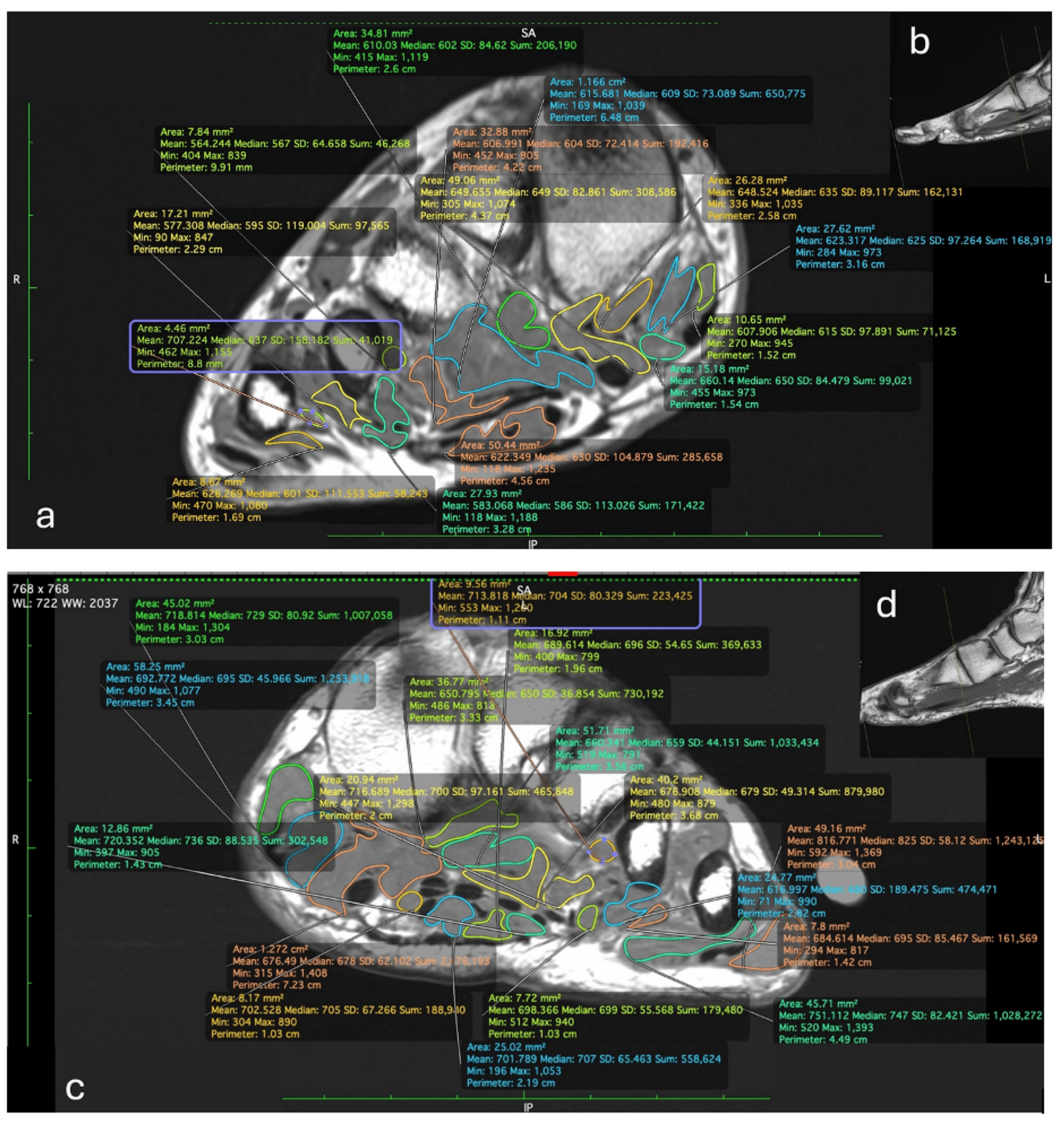
Foot muscle segmentation on coronal magnetic resonance imaging (MRI) of a healthy patient (a) and a patient with a history of falls (c). Measurement level at the first tarsometatarsal joint was confirmed on sagittal views (b and d). Segmented areas were summed to determine the total muscle cross-sectional area.

All measurements were performed on a short-axis (Coronal) anatomic sequence (either proton density or T1-weighted) at the level of the first tarsometatarsal joint. The anatomical location for the measurement was confirmed using the 3D position tool and the dorsal aspect of the first tarsometatarsal joint on axial (long axis) images as a reference point (Figure 1).

The freehand region of interest (ROI) tool was employed for muscle segmentation, automatically calculating the area for each segment. The total area was then obtained by manually summing the segment areas. Due to the challenge of accurately identifying individual muscles, particularly the smaller ones, all foot musculature on the images was included in the ROI. Non-contractile components, including bone, tendon, and fat, were excluded based on their distinct signal intensity compared to muscle tissue, ensuring that only the contractile muscle components were quantified for analysis.

Statistical analysis

Statistical analysis was performed using IBM SPSS Statistics for Windows, Version 28 (Released 2021; IBM Corp., Armonk, New York, United States). Baseline characteristics of the study groups were compared using the independent t-test for continuous variables and the chi-square test for categorical variables. Continuous variables are reported as mean ± standard deviation (SD), while categorical variables are expressed as percentages. Inter-rater reliability for CSA measurements was assessed using the intraclass correlation coefficient (ICC), with values categorized as poor (<0.5), moderate (0.5-0.75), good (0.75-0.90), and excellent (> 0.90) [17]. Multiple logistic regression analysis was conducted with study group membership as the dependent variable, incorporating muscle CSA and other covariates as independent variables. Odds ratios (ORs) with their corresponding 95% confidence intervals (CIs) were reported. Additionally, collinearity was assessed using the Variance Inflation Factor (VIF) with a cutoff of 3 indicating high collinearity [18]. A post hoc power analysis was conducted using G*Power software and CSA as the primary outcome. The analysis demonstrated an effect size of 1.16, with a two-tailed Type I error probability of 0.05, yielding a statistical power of 0.79. Statistical significance was defined as p > 0.05.

## Results

A total of 13 cases aged 70.6 ± 8.5 years and 13 controls aged 69.5 ± 6.9 were included in the study. The comparison of the baseline characteristics did not reveal any differences except for CSA measurements (P=0.006; Table 1).

**Table 1:** A comparison of the baseline characteristics of the study groups. Values are presented in percentage or mean ± SD.

Characteristics	Cases	Controls	P-value
Age	70.62 ± 8.5	69.46 ± 6.9	0.7
Body mass index*	26.7 ± 6.3	27.36 ± 3.7	0.7
Gender (Female)	38% (n=5)	38% (n=5)	1
Race (White)	67% (n=8) †	84% (n=11)	0.29
Lower extremity pain	69% (n=4)	70% (n=4)	0.99
Polypharmacy (≥5 medications)	53% (n=7)	69% (n=9)	0.42
Walking aids	30% (n=4)	7.6% (n=1)	0.13
Peripheral vascular disease	7.6% (n=1)	7.6% (n=1)	1
Cross-sectional area**	285.5 ± 220.2	542 ± 218.6	0.006
* Kilogram per square meter ** Square millimeter † Data was missed for one patient			

The ICC measures showed excellent agreement between the two raters for the CSA measurements (ICC=0.92, 95% CI: 0.82, 0.96, P-value < 0.001). The association between study group membership and foot muscle CSA was significant, with cases showing a lower CSA (OR=0.99, 95% CI: 0.98, 0.99), P = 0.03), even after adjusting for the effects of other variables. The remaining variables did not demonstrate statistically significant associations with CSA measurements (Table 2).

**Table 2:** Multivariable linear regression analysis of factors associated with recurrent falls. Odds ratios, their associated 95% confidence intervals (CIs), and collinearity statistics are presented.

Variables*	Odds Ratio (95% CI)	P-value	Collinearity Statistics (VIF**)
Foot muscle CSA †	0.99 (0.98, 0.99)	0.03	1.14
Race (White=1, non-White=0)	2.26 (0.06, 85.32)	0.66	1.26
Body Mass Index	0.88 (0.69, 1.12)	0.32	1.31
Lower extremity pain (Yes=1, No=0)	2.09 (0.14, 31.26)	0.59	1.12
Polypharmacy (Yes=1, No=0)	0.10 (0.006, 1.96)	0.13	1.06
Walking aids (Yes=1, No=0)	11.46 (0.20, 646.03)	0.23	1.45
Peripheral vascular disease (Yes=1, No=0)	0.36 (0.002, 55)	0.69	1.13
* The coefficients for categorical variables are reported for the subgroups assigned “1” (as indicated in parentheses) ** Variance Inflation Factor † Cross-sectional Area			

VIF values indicated no significant multicollinearity between the variables.

## Discussion

Foot muscle strength is diminished in individuals with recurrent falls. While radiological measurements of foot muscle size correlate with muscle strength, their association with recurrent falls remains unexplored. Our preliminary results showed the feasibility of using MRI to measure CSA of foot muscles in older individuals and identified an association between CSA and a history of recurrent falls.

Several measures have been described to quantify foot muscle strength without consensus on the ideal measurement technique. Direct measures of muscle strength, such as dynamometry, are accurate; however, they lack the specificity to differentiate between the strength of the intrinsic and extrinsic musculature [19,20]. Therefore, the use of indirect imaging has gained attention, with MRI often regarded as the reliable modality [21]. Early cadaver studies in the lower limb demonstrated a high correlation between MRI-derived muscle CSA and direct cadaveric measurements [22]. Later, clinical studies have found a correlation between toe flexion strength measured directly with a dynamometer to the CSA of intrinsic and extrinsic foot musculature as measured by MRI in young healthy adults [12,23]. These findings confirmed the feasibility of using MRI measures as a marker of muscle strength in the young healthy adult population.

While the reasons for falls in the elderly are complex and multifaceted, the presence of reduced muscle strength may contribute to toe deformities that contribute to falls [8-10,24,25]. Age-related sarcopenia generally affects muscle strength throughout the body; decreased foot muscle strength may serve as an indicator of the broader scope of sarcopenia, highlighting the importance of comprehensive physical assessments in the elderly population [19,26]. While the evidence supports the use of radiologic surrogates of muscle strength, their independent association with incident falls in older adults is unclear. In the present study, each standard deviation (6.9 cm^2^) increase in MRI-derived muscle CSA was associated with 1% lower odds of recurrent falls (OR=0.99, 95% CI [0.98, 0.99], P=0.03), independent of BMI, lower-extremity pain, polypharmacy, and walking aids. These findings align with the previous literature that interventions aimed at increasing plantar intrinsic muscle strength improve fall-related dynamic function and balance in older adults [27-30]. Furthermore, they demonstrate a possible role for CSA measurements in fall risk assessment.

While this study adds to our understanding of those at risk for recurrent falls, it has limitations. First, the study is limited by a small study population size. This was a result of the narrow inclusion criteria necessary to account for confounders in this retrospective case-control study design. Accordingly, the findings should be viewed as hypothesis-generating and warrant confirmation in larger, prospective cohorts. Furthermore, due to the retrospective nature of the study, we could not account for the heterogeneity in the MRI images, which might introduce bias to the measurements. This also hindered individual muscle CSA measurements, and a summation of all plantar musculature was calculated instead. Although previous studies have identified specific plantar muscle groups most associated with toe flexion strength, we cannot isolate differences to specific muscle groups in those with falls as compared to those who did not have a history of falls.

## Conclusions

The present study highlights the possible role of morphological changes in foot muscles on MRI in patients with recurrent falls. Our results provide a foundation for future validation studies with larger sample sizes to explore the utility of imaging assessment to enhance fall prevention strategies and enable more targeted interventions
